# Automated Fiber Placement Path Planning and Analysis of Pressure Vessels

**DOI:** 10.3390/ma16186187

**Published:** 2023-09-13

**Authors:** Bo Wang, Lihua Wen, Jinyou Xiao, Shiyu Wang, Ping Ren, Liqiang Wang, Lei Zu, Xiao Hou

**Affiliations:** 1 School of Astronautics, Northwestern Polytechnical University, Xi’an 710072, China; wang_bo@mail.nwpu.edu.cn (B.W.); xiaojy@nwpu.edu.cn (J.X.); wangsy@nwpu.edu.cn (S.W.); houxiaoht@163.com (X.H.); 2 Xi’an Institute of Aerospace Propulsion Technology, Xi’an 710025, China; 3 The 41st Institute of the Fourth Academy of China Aerospace Science and Technology Corporation National Key Lab of Combustion, Flow and Thermo-Structure, Xi’an 710025, China; 4 School of Mechanical Engineering, Hefei University of Technology, Hefei 230000, China; zulei@hfut.edu.cn

**Keywords:** composite pressure vessel, automated fiber placement, placement path planning, graphic visualization

## Abstract

The automated fiber placement (AFP) process faces a crucial challenge: the emergence of out-of-plane buckling in thermoplastic prepreg tows during steering, significantly impeding the quality of composite layup. In response, this study introduces a novel approach: the development of equations for wrinkle-free fiber placement within composite pressure vessels. The investigation encompasses a detailed analysis of prepreg trajectories in relation to shell geometry, accompanied by an in-depth understanding of the underlying causes of wrinkling on dome surfaces. Moreover, a comprehensive model for shell coverage, grounded in placement parameters, is meticulously established. To validate the approach, a simulation tool is devised to calculate press roller motions, ensuring the uniform fiber dispersion on the mandrel and achieving flawless coverage of the shell without wrinkles. This innovative strategy not only optimizes the AFP process for composite layup but also remarkably enhances the overall quality of composite shells. As such, this research carries significant implications for the advancement of composite manufacturing techniques and the concurrent improvement in material performance.

## 1. Introduction

Composite pressure vessels have been utilized for several decades as a lightweight solution for containing gas or fluid under pressure, being widely used in aerospace, military, and aquatic sector industries [1,2]. The cutting-edge technology of automated fiber placement (AFP) has revolutionized the fabrication of diverse composite products, including large storage tanks, pipeline systems, pressure vessels, and rocket motor casings. Different from other methods [3,4], AFP does not rely on tension to fix the prepreg position, but instead, it cooperates with the robot’s movement to create the prepreg placement path. Moreover, AFP’s in situ technology can effectively enhance the molding rate of products without relying on secondary curing molding equipment, which can reduce the equipment investment and decrease the product production cycle. Due to its myriad benefits, AFP stands as the progressive direction in pressure vessel preparation [5,6,7]. The generation of the placement path is one of the most crucial processes in the placement process, which including placement path generation [8,9,10,11], placement path evaluation [12,13,14,15,16], and motion simulation [17,18,19]. An ideal placement path can effectively prevent the occurrence of wrinkle defects during the prepreg-forming process. This can be achieved by placing fiber tows onto the mandrel surface of a desired shape with designed placing patterns to ensure uniform fiber distribution, to prevent defect accumulation, and to enhance the mechanical properties of composite pressure vessels.

In recent years, many researchers have investigated the placement path planning of AFP. Belnoue et al. [20] introduced the formation of out-of-plane wrinkles in the debulking and autoclave curing processes of laminates with embedded gaps and overlaps between the deposited tapes. Predictions were made using a novel modeling framework and validated against micro-scale geometry characterization of artificially manufactured samples. This study provided valuable insights to guide defect modeling within AFP processes. Christopher et al. [21] utilized finite element simulation and an optimization algorithm to design the prepreg placement path of a flat structure and employed the sensitivity optimization method of the level set function to plan the prepreg placement path of the flat structure. The simulation results showed that this approach effectively increased the laminate’s stiffness by 41.5% and 23.4% under two boundary conditions. Bijan et al. [22] proposed a novel uniform fiber placement path planning algorithm for robotics. The algorithm formulates a set of surface curves representing the path of the composite sheet to ensure the uniform laying of subsequent streamers without gaps or overlaps. The algorithm was a numerical validation and an industrial implementation that substantiated the algorithm’s efficacy. Cong et al. [23] evaluated the laying quality of the fiber trajectory by examining the tow deformation characteristics of prepreg on freeform surfaces and suggested an AFP path evaluation algorithm based on prepreg tow deformability. Pierre et al. [24] presented a tool path smoothing method applied to the AFP process. The smoothing method aims to minimize the curvature and rotational axis order variations. The algorithm was based on a robust filtering method that ensures a short computing time, was adapted to redundant machine tool, and finally took into account the difference in the dynamic characteristics of each axis in the objective function to generate the fastest tool path. Qu et al. [25] contributed a wrinkle defect criterion for prepreg forming on curved surfaces, capitalizing on the analysis of the filament deformation characteristics of prepreg on free-form surfaces. As the results show, the laying of curved parts demonstrated that placement path planning incorporating this criterion could effectively suppress defects such as wrinkles and bridging.

The mechanical properties of composite pressure vessels are greatly affected by the prepreg laying angle, making placement path planning crucial in their manufacturing process. The prepreg is shaped in situ on the mandrel surface according to the planned path under the action of the press roller mechanism, with a specific bond strength required between the layers. However, limitations in the AFP technology and the prepreg’s forming characteristics could result in an unsuitable placement path that does not meet the design requirements. To reduce the need for costly modifications during placement path planning, this study introduces an innovative approach that harmonizes the prepreg suitability analysis with pressure vessel placement readiness. Specifically, this paper incorporates the wrinkle-free laying trajectory criterion of prepreg on curved surfaces with the shell surface curve equation to derive the laying trajectory equations that comply with the process requirements. This approach maximizes the utilization of the composite material strength and expands the design space of laying patterns. Ultimately, the proposed method offers invaluable insights into the preparation of pressure vessels with irregular pole holes and streamlines the efficiency of placement path planning.

## 2. Placement Path Planning of Pressure Vessel

The pressure vessel is composed of two domes at each end and a cylinder section in the middle. Both ends have different geometric forms that require distinct considerations for the design of the placement path. As a result, equations for the placement path are derived with slight variations to account for the differences in geometrical parameters between the two sections.

### 2.1. Placement Path Planning for Wrinkle-Free Defects in the Ellipsoidal Dome Section

**Figure 1:**
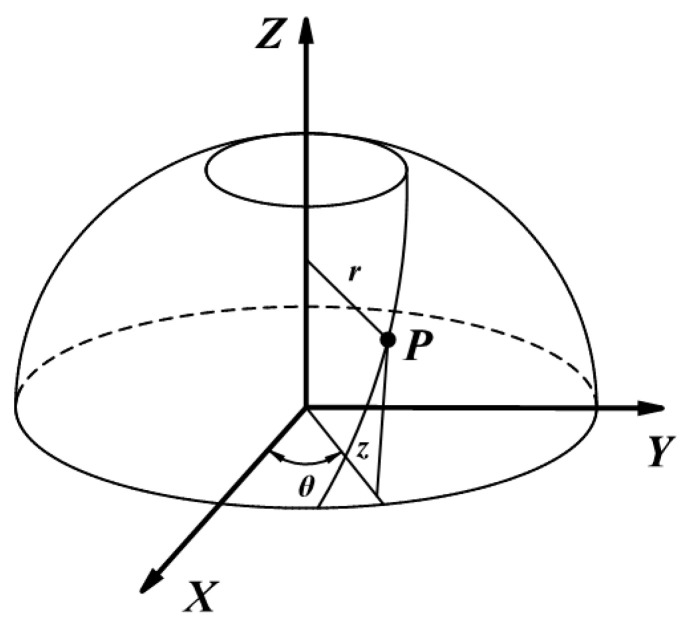
Placement path on a surface of revolution.

Figure 1 depicts that ellipsoidal dome sections can be modeled as a curved surface that rotates counterclockwise around the Z axis. The pressure vessel’s surface can be described using the cylindrical coordinate system {r, θ, z}, and the relevant constitutive equations are shown as follows:

(1) r(θ,z)=(rcosθ,rsinθ,Z)

where r and z represent the radius and axial distance of the rotary body, and θ is the mandrel rotation angle.

In order to prevent the occurrence of wrinkle defects resulting from the accumulation of defects during prepreg placement on a curved surface, Qu et al. [25] investigated the strain variation of prepreg during surface placement of a specific width and established a criterion condition for wrinkle-free placement of prepreg on the curved surface.

(2) $$\left|\frac{W}{2}\times K_{g}-\frac{W^{2}}{8}\times K\right|\leq \frac{W}{2\times R_{min}}$$

where W is the width of the prepreg; $K_{g}$ is the geodesic curvature of the placement path; K is the Gaussian curvature of the surface; and $R_{min}$ is the minimum forming radius at which the prepreg can be laid on a flat surface without wrinkle defects.

From the Liouville formula for differential geometry, the geodesic curvature $K_{g}$ of the curve on the surface of the slalom and the Gaussian curvature K of the surface can be expressed as follows:

(3) $$K_{g}=\frac{d\alpha }{ds}+\frac{r^{\prime }\sin\alpha }{r\sqrt{1+{r^{\prime }}^{2}}}$$

(4) $$K=\frac{-r^{\prime \prime }}{r{\left(1+{r^{\prime }}^{2}\right)}^{2}}$$

where α is the angle between the curve on the surface and the surface meridian, called the laying angle; s is the coordinate of the corresponding arc length of the curve; $r^{\prime }$ is the derivative of r with respect to z; and r″ is the second derivative of r with respect to z. Meanwhile, the parameters z, s, α, θ, and r′ on the surface of the slalom satisfy the following differential equation relations [26,27]:

(5) $$\frac{dz}{ds}=\frac{\cos\alpha }{\sqrt{1+{r^{\prime }}^{2}}}$$

(6) $$\frac{d\theta }{dz}=\frac{\tan\alpha \sqrt{1+{r^{\prime }}^{2}}}{r}$$

Since the Gaussian curvature of the surface and the prepreg width W is constant values, Equation (2) expresses the relationship between $K_{g}$ and $R_{min}$ in the absence of wrinkle defects in the prepreg. The absolute value exists on Equation (3)’s left-hand side. Hence, two cases exist. In case I, ($K_{g}-\frac{W^{2}}{8}*K\geq 0$), when the prepreg is laid on the surface, the strain reaches a critical state, and Equation (3) is deformed as follows:

(7) $$K_{g}=\frac{1}{R_{min}}+\frac{W\times K}{4}$$

Similarly, in case II, ($\frac{W}{2}*K_{g}-\frac{W^{2}}{8}*K<0$), Equation (2) is deformed as follows:

(8) $$K_{g}=\frac{W\times K}{4}-\frac{1}{R_{min}}$$

According to Equations (7) and (8), the geodesic curvature of the without wrinkle placement path on the surface with constant geometry and prepreg width depends on $R_{min}$. As $R_{min}$ tends toward infinity, Equations (7) and (8) can be calculated to obtain the range of values of $K_{g}$. Moreover, according to the combination of Equations (3)–(6), Equations (7) and (8)’s deformation can obtain Equations (9) and (10) as follows:

(9) $$\frac{d\alpha }{dz}=\frac{\sqrt{1+{r^{\prime }}^{2}}}{R_{min}\cos\alpha }+\frac{-Wr^{\prime \prime }\sqrt{1+{r^{\prime }}^{2}}}{4r{\left(1+{r^{\prime }}^{2}\right)}^{2}\cos\alpha }-\frac{r^{\prime }}{r}\tan\alpha$$

(10) $$\frac{d\alpha }{dz}=\frac{Wr^{\prime \prime }\sqrt{1+{r^{\prime }}^{2}}}{4r{\left(1+{r^{\prime }}^{2}\right)}^{2}\cos\alpha }-\frac{\sqrt{1+{r^{\prime }}^{2}}}{R_{min}\cos\alpha }-\frac{r^{\prime }}{r}\tan\alpha$$

In the case of laying the prepreg on the dome of the pressure vessel without wrinkle defects, the differential equation relationship for the change in laying angle along the axial direction is given by Equations (9) and (10). These are non-linear differential equations, which can be solved numerically using the Runge–Kutta formula to determine the range of variation in α along the dome axis, given the geometrical parameters of the pressure vessel and the width of the prepreg.

**Figure 2:**
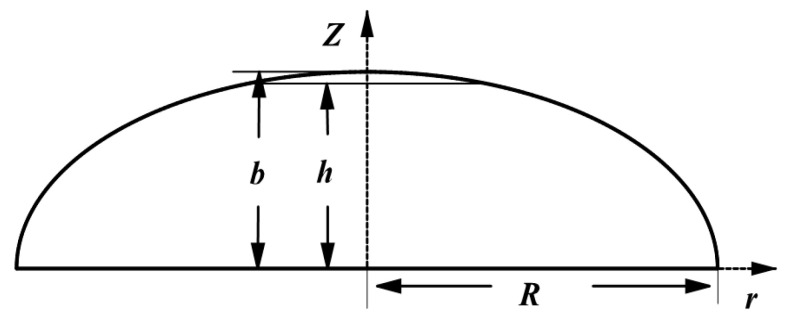
Geometry of an ellipsoidal dome.

**Figure 3:**
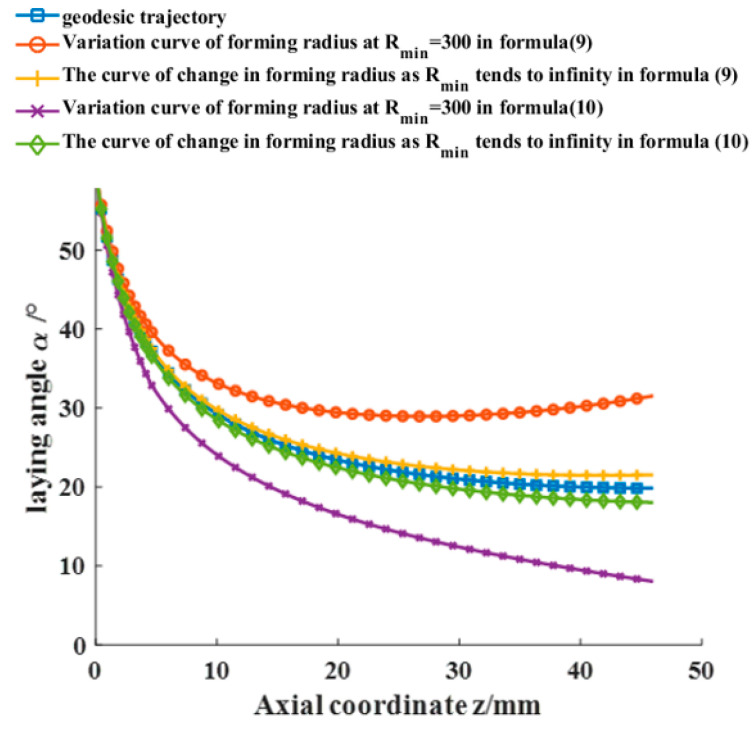
Range of laying angle for the prepreg place placement without wrinkle defects in the left ellipsoidal dome section.

**Figure 4:**
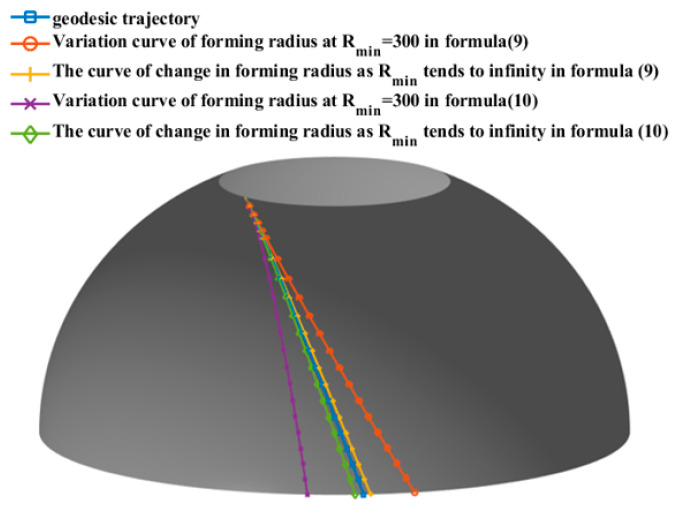
Actual placement path of the ellipsoidal dome section.

To verify the universality of placement path planning, this study selects an unequal pole hole pressure vessel as the object of study. The meridian curve equation is $\frac{z^{2}}{b^{2}}+\frac{r^{2}}{R^{2}}=1$, where b and R are the semi-minor and semi-major axes, respectively, of the ellipse with values of 50 mm and 53 mm, as shown in Figure 2. The left and right head heights are h1 = 46 mm and h2 = 42 mm, respectively; the cylindrical length is L = 200 mm; the prepreg width is W = 6.35 mm; and $R_{min}$ = 300 mm. The initial laying angle of the left dome is set to 60°. By applying Equations (9) and (10), the end laying angle variation of prepreg without wrinkle defects of the left ellipsoidal dome near the cylinder section is calculated to be [8.03°, 18.04°] ∪ [21.54°, 31.55°], and the laying angle variation along the axial direction of the ellipsoidal dome is shown in Figure 3. Combined with Equation (6), the Runge–Kutta formula is used to calculate the mandrel rotation angle θ of the placement path under the corresponding laying angle, and the three-dimensional placement path of the ellipsoidal dome is shown in Figure 4. As the minimum forming radius $R_{min}$ tends toward infinity (with a value of $10^{25}$ mm selected as infinite in this paper), the placement path of the dome section will approximate the geodesic curve with an initial angle of 60° but will not coincide with it, as shown in Figure 3 and Figure 4. The analysis of Equations (9) and (10) indicates that the influence of the Gaussian curvature of the dome surface and the geodesic curvature of the placement path makes it impossible to place the prepreg on certain geodesic lines on the surface without wrinkle defects, regardless of the value of $R_{min}$, which also explains why, as described in the literature [28], prepregs with a certain width may sometimes produce wrinkle defects even when placed on the geodesic path of a curved surface. The wrinkle-free laying angle range at an ellipsoidal dome section is enlarged by reducing the $R_{min}$ value of a preform plane placement, the selection of deployment placement path parameters at the ellipsoidal dome section is expanded, and the optimal deployment placement path parameters are subsequently optimized to use an optimization algorithm and a finite element analysis.

### 2.2. Placement Path Planning for Defect-Free Algorithm in the Ellipsoidal Dome Section

For composite plies formed using AFP, gaps and overlaps can frequently occur between two adjacent prepregs in the same ply due to the mandrel’s shape and placement path planning algorithm. These gaps and overlaps have negative impacts on the entire placement process and the structural integrity of composite layers, including reduced laying efficiency and increased material losses due to more shearing and re-feeding operations of the filament bundle. Moreover, the gaps and overlaps can affect the uniformity of layer thickness and the dimensional accuracy of the composite component shape, and also result in mechanical property variations at different locations, leading to damage and failure of the layers where gaps exist.

**Figure 5:**
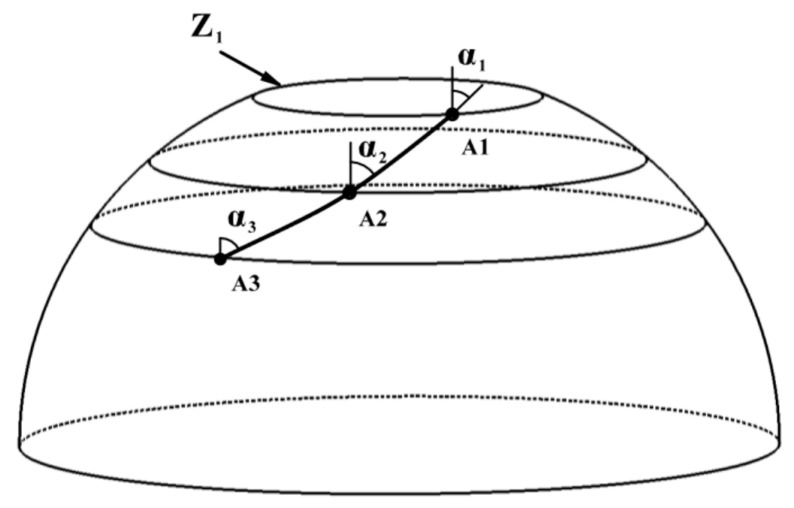
Schematic diagram of the prepreg placement path on the dome surface of the pressure vessel.

To address these challenges, a defect-free placement path planning algorithm is proposed for the dome section that combines wrinkle-free placement paths with prepreg and dome geometry to achieve gap-free, overlap-free, and wrinkle-free placement of the prepreg. Pressure vessels are rotary structures characterized by a straight axis, a circular interface perpendicular to the axis, and a spline curve bus. To illustrate the specific implementation steps of the no defect placement path planning algorithm for the ellipsoidal dome of the pressure vessels, the schematic depiction of a pressure vessel is showcased in Figure 5 to elucidate the precise steps involved in the defect-free placement path planning algorithm.

The prepreg placement path in the ellipsoidal dome can be discretized into m points (A1, A2, …, Am); a plane perpendicular to the axis is made over each point; and the resulting plane is defined as the cross-section of the pressure vessels with the cross-section circle corresponding to a perimeter of $C_{m}$, where the cross-section circle at the pole hole of the ellipsoidal dome is the smallest and the corresponding perimeter is $C_{1}$.

**Figure 6:**
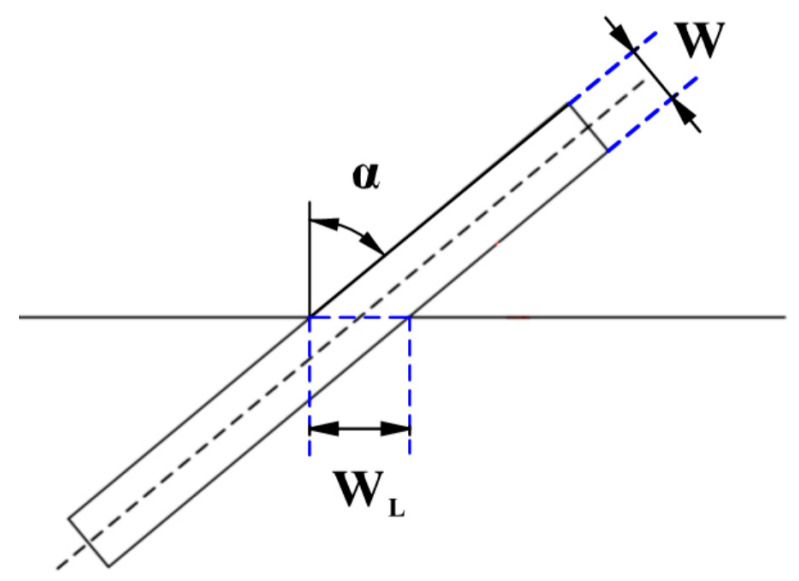
Schematic diagram for calculating the actual cover width for prepreg laying.

The crux of designing an ellipsoidal dome section with no defects lies in accurately determining the laydown angle of the prepreg. Figure 6 illustrates that the width covered by the prepreg at the laydown angle α is denoted as $W_{L}$.

(11) $$W_{L}=\frac{W}{\cos\alpha }$$

Based on the analysis of the actual coverage width of the prepreg described above, it is feasible to achieve continuous, defect-free placement of prepreg along a certain laying angle without any gaps or overlapping defects. This approach is now being applied to the mandrel surface. As the mandrel cross-section undergoes continuous change, the laying angle of the prepreg can also be adjusted accordingly for the purpose of forming a placement path that is free of gaps and defects, with continuously changing laying angles. The design of this placement path can be divided into three steps. The first step involves setting the initial laying angle and calculating the number of laps of prepreg laying. For example, when studying the left ellipsoidal dome, the pole hole with the smallest circumference of the section is designated as the starting point of laying, the initial laying angle is set to $\alpha_{1}$, and the number of laps of laying N is calculated.

(12) $$N=\left\lceil \frac{2\pi r_{Z_{1}}}{WL}\right\rceil$$

where $r_{Z_{1}}$ is the radius of the cross-sectional circle at the pole hole.

The second step involves correcting the actual initial forming angle $\alpha_{c}$ and calculating the laying angle of the placement path. The number of laps N required to achieve even and defect-free coverage of the dome region with prepreg is determined through Equation (12). As N is obtained by rounding up, further correction and calculation are required to determine the actual initial laying angle $\alpha_{c}$.

(13) $$\alpha_{c}=\sin^{-1}\left(\frac{WN}{2\pi r_{Z_{1}}}\right)$$

Similarly, the laying angle $\alpha_{m}$ at the other intersections of the placement path can be expressed as follows:

(14) $$\alpha_{m}=\sin^{-1}\left(\frac{WN}{2\pi r_{Z_{m}}}\right)$$

**Figure 7:**
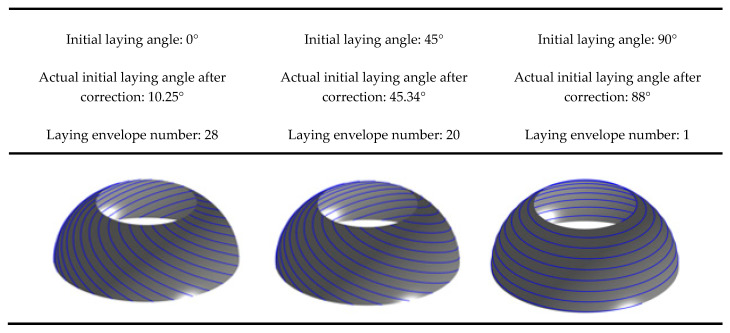
Defect-free ellipsoidal dome sections’ placement path pattern.

**Figure 8:**
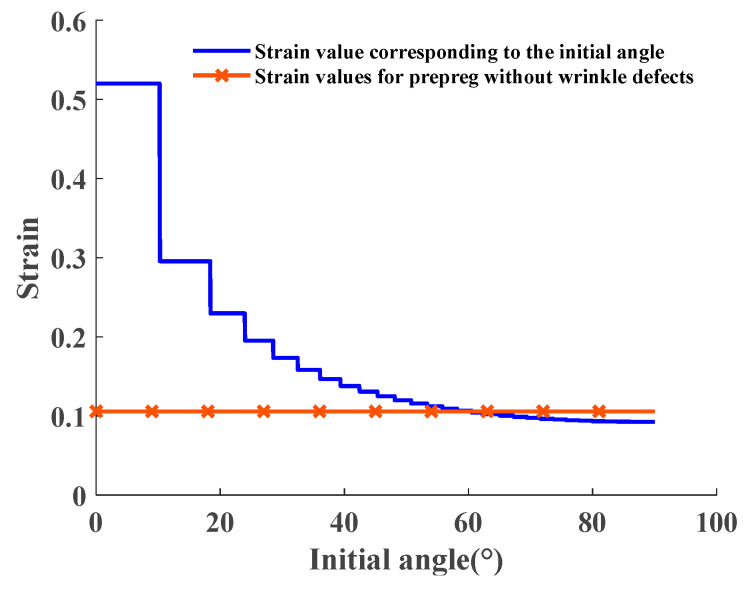
Variation in strain in the placement path of the ellipsoidal dome regions with variable initial laying angle.

The third step involves conducting a wrinkle detection analysis of the placement path. By combining the wrinkle-free criterion, the placement path obtained in the second step is evaluated for the presence of wrinkle defects. Taking the right ellipsoidal dome of the mandrel as an example, the minimum forming radius $R_{min}$ is set to 30 mm for the prepreg plane lay-up without wrinkle defects. With the initial laying angle analysis range set to [0°, 90°], the maximum prepreg strain corresponding to different initial lay-up angle paths of the right ellipsoidal dome is calculated by using Equations (2)–(4) and compared with the selected prepreg limit strain without wrinkle defects. As depicted in Figure 7, since corrections must be made according to the number of placement paths after a given initial laying angle, a specific range of initial lay-up angles corresponds to the same actual initial laying angle and placement path. Hence, step-like fold strain values will appear in Figure 8. Based on the calculation results, the range of initial angle selection for the right ellipsoidal dome without defects is determined to be [60.56°, 90°]. As the initial angle of the laying curve increases gradually, the variation in strain values during the placement of prepreg exhibits a progressively gentle trend. Furthermore, the placement path pattern of the ellipsoidal dome depicted in Figure 7 can also be employed for reinforcement structures of the pressure vessels. The construction of ellipsoidal dome structures using a multi-layered deposition approach enables precise reinforcement of vulnerable regions within the pressure vessels. This precise reinforcement leads to an effective enhancement in the strength utilization efficiency of the fiber material, concurrently reducing the superfluous mass of the vessels. This reinforcement strategy not only contributes to the optimization of structural performance in pressure vessels but also unveils broader prospects for practical engineering applications.

By means of simulation analysis and verification, the proposed defect-free placement path calculation method for the dome section of the mandrel structure, which integrates the analysis of mandrel and prepreg geometry and incorporates the prepreg wrinkle criterion, has been demonstrated to be highly effective in guiding the prepreg to achieve gap-free, overlap-free, and wrinkle-free placement in the dome section.

### 2.3. Placement Path Planning for Wrinkle-Free Defects in the Cylinder Section

The cylinder section is a rotary structure with equal sections, characterized by K, r′, and r″ values of 0. By combining Equations (9) and (10) with the geometrical characteristics of the cylinder section, the wrinkle-free placement path equation for the cylinder section is analytically derived as follows:

(15) $$\frac{d\alpha }{dz}=\frac{1}{R_{min}\cos\alpha }$$

(16) $$\frac{d\alpha }{dz}=-\frac{1}{R_{min}\cos\alpha }$$

**Figure 9:**
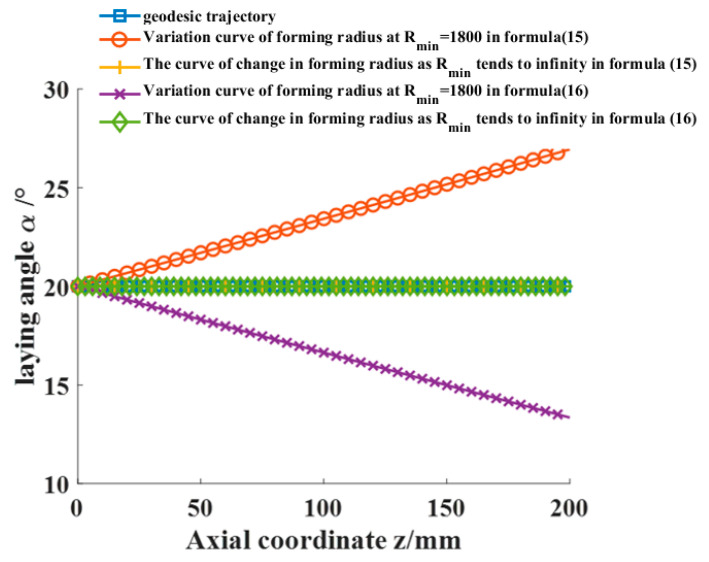
Range of angles for laying prepreg without wrinkle defects in the cylinder section.

**Figure 10:**
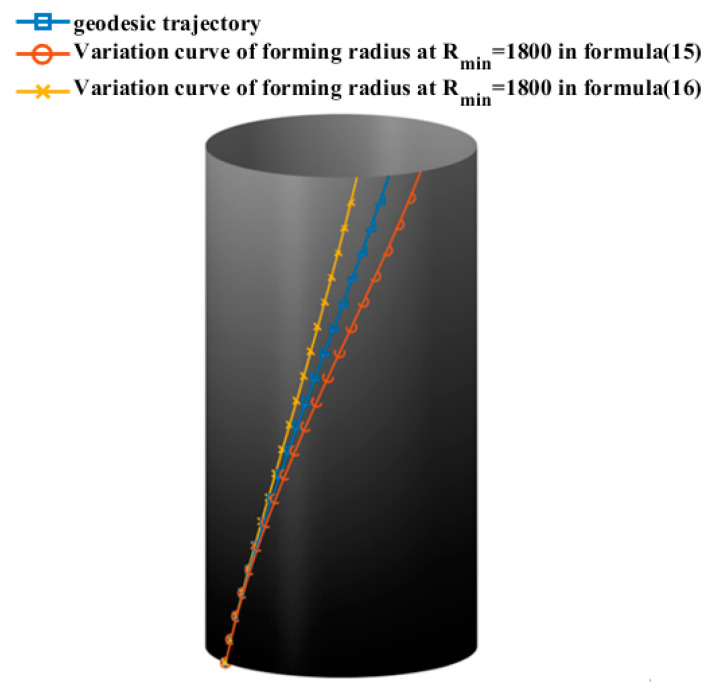
Placement path in the cylinder section.

Using the aforementioned cylinder section as an example, with the initial laying angle set at 20° and the minimum forming radius $R_{min}$ = 1800 mm of the prepreg adjusted, the range of prepreg laying angles that result in wrinkle-free placement for the cylinder section is calculated using Equations (15) and (16) as [13.35°, 26.94°]. The axial variation of the laying angle is illustrated in Figure 9, while the actual placement trajectory of the cylindrical portion is depicted in Figure 10. Equations (15) and (16) serve as the theoretical underpinnings for the AFP process designed for variable-angle placement paths devoid of any wrinkle defects on the surface of the cylinder. These equations establish the foundational framework for achieving precise and controlled fiber deposition on the cylindrical surface, ensuring the production of defect-free composite structures. Their seamless integration into the fabrication procedure facilitates the realization of optimized fiber configurations, ushering in amplified mechanical properties and heightened structural integrity within the resultant composite materials.

As the minimum forming radius of the prepreg $R_{min}$ approaches infinity, the wrinkle-free placement path of the cylinder section converges to the geodesic curve. This implies that a prepreg with a certain width can be placed on the surface of a rotating body with a constant cross-section without any wrinkle defects, following the geodesic trajectory.

Deforming and integrating Equations (15) and (16) give

(17) $$\int_{0}^{L}dz=\pm \int_{\alpha_{0}}^{\alpha }R_{min}\cos\alpha d\alpha$$

The equation for the axial length corresponding to the change in the laying angle of the cylinder section is obtained:

(18) $$L=\left|R_{min}(\sin\alpha -\sin\alpha_{0})\right|$$

where $\alpha_{0}$ is the initial laying angle, α is the termination laying angle, and L is the axial length of the cylinder corresponding to the without-wrinkle-defect lay of the prepreg required for the transition between the two laying angle changes.

## 3. Full Coverage

To ensure uniform coverage of the mandrel with prepreg without wrinkle defects, the prepreg laying process starts from the left head pole hole, extends to the right pole hole, and returns. A complete cycle involves staggering the prepreg width at the cylinder equator, followed by multiple cycles to achieve full coverage. However, not all mandrel rotation angles allow for even filling, but rather only when the mandrel rotation angle reaches a specific value can conditions for even filling be met.

During the AFP process, the mandrel rotates one cycle of roundtrip movement of the press roller, denoted by ∑θ. The total mandrel rotation angle can be expressed as follows:

(19) $$\sum \theta =\theta_{a}+\theta_{b}+∆\theta =360\;(M\pm \frac{k}{Y})$$

where $\theta_{a}$ and $\theta_{b}$ represent the ellipsoidal dome and cylinder section placement path corresponding to the mandrel rotation angle, respectively; ∆θ is a tiny angle; and M is the number of revolutions of the mandrel when the prepreg is covered. Y is similar to N in Equation (12) and represents the number of cycles required for the mandrel to be uniformly covered by prepreg. The laying angle α involved in the calculation of the actual laying coverage width is the minimum laying angle of the cylinder section. K is an integer number that satisfies the requirement that k/Y is the simplest true fraction.

**Figure 11:**
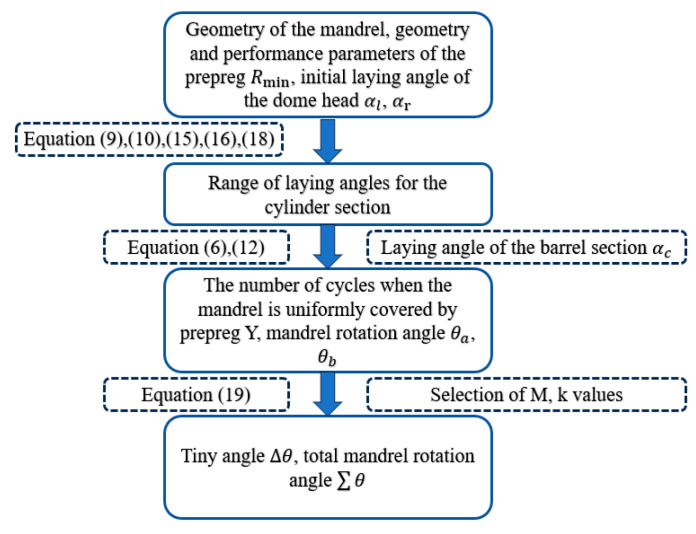
Flow chart of the optimal design procedure for determining the placement process parameters.

**Figure 12:**
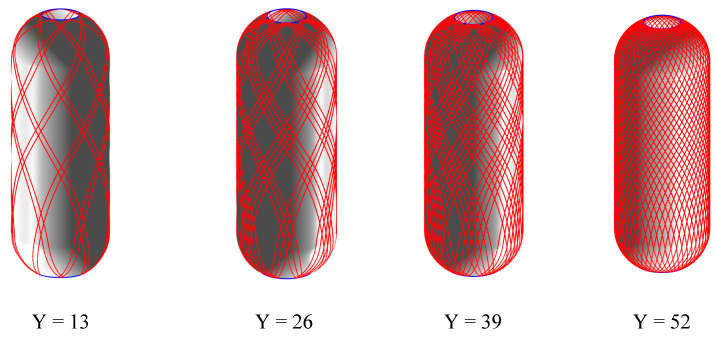
Placement path patterns for the Y = 13, Y = 26, Y = 39, and the final placed circuit.

The process of analyzing the full coverage of the placement path is illustrated in Figure 11. The mandrel and prepreg described above are analyzed for placement path coverage. The prepreg minimum forming radius is set to $R_{min}$ = 300 mm. The initial laying angles of the left and right ellipsoidal domes are 60° and 30°, and the final laying angles of the left and right ellipsoidal domes are calculated to be within the range of [8.03°, 18.039°] ∪ [21.54°, 31.55°] and [5.71°, 14.19°] ∪ [17.27°, 26.03°], respectively. The final laying angles of the left and right ellipsoidal domes are set as 25° and 10°, respectively, to obtain a constant laying angle range of [0°, 39.15°] for the cylinder section. A constant laying angle of 30° is selected for the cylinder section. The value of M is chosen as 2, and the value of k is chosen as 37, resulting in a tiny angle of ∆θ = 97.39°. Based on the above data, the effect of prepreg forming for different laying paths is shown in Figure 12. The minimum laying angle for the cylinder section is 10°, and the minimum coverage fraction of prepreg on the cylinder section is as follows:

$$T=\frac{WY}{2\pi R\cos\;\alpha }\times 100\%=\frac{6.35\times 52}{106\pi \cos\left(10^{{}^{\circ}}\right)}=100.69\%$$

In the actual placement of prepared pressure vessels, it is acceptable for the coverage fraction to be greater than 100% due to the parameter Y in the cylinder section typically being rounded upwards.

## 4. Motion Control Analysis for the Placement Path of Pressure Vessel

**Figure 13:**
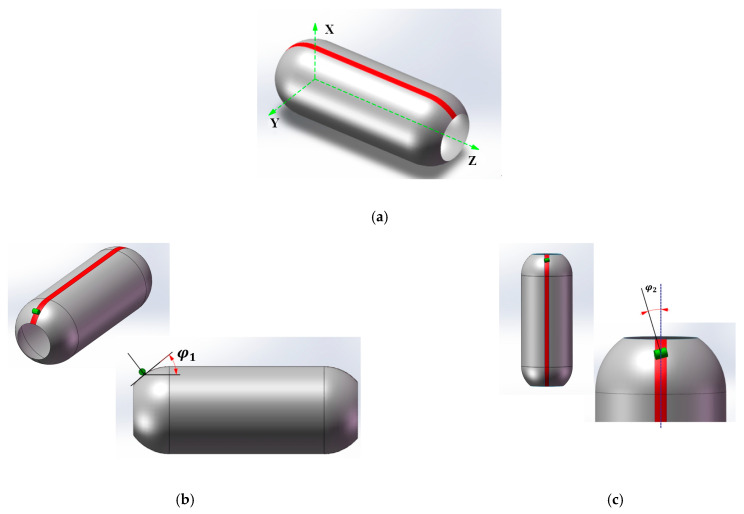
The coordinate system for the layering head motion and its trajectory on the vessel surface: (**a**) the movement zone; (**b**) the angle $\varphi_{1}$ and (**c**) the angle $\varphi_{2}$.

Based on the results of the placement path solution, the coordinate position of the prepreg laying point can be determined. However, to validate the accuracy of the placement path planning method, it is essential to conduct actual laying verification on the machine, involving examining the trajectory of the press roller and the mandrel rotation axis. During the prepreg placement process on the mandrel surface for pressure vessel preparation, the press roller mechanism is only required to move within the red area illustrated in Figure 13a to ensure complete coverage of the pressure vessel.

During the preparation of pressure vessels using AFP, the accuracy of the prepreg’s laying angle plays a crucial role in determining the accuracy of the placement path. The press roller is responsible for controlling the laying angle, and as the mandrel rotates, the press roller moves axially within the red area of the mandrel surface. The laying angle of the prepreg is precisely controlled by regulating the $\varphi_{1}$ and $\varphi_{2}$ angles of the press roller and mandrel, respectively. In Figure 13b, $\varphi_{1}$ represents the angle between the projection of the contact plane of the press roller and the mandrel in the XZ plane and the YZ plane. In Figure 13c, $\varphi_{2}$ takes on the mantle of the angle linking the press roller’s radial direction with the mandrel’s axial trajectory—a magnitude synonymous with the prepreg’s laying angle α within the contours of placement path analysis.

From Figure 13b, $\varphi_{1}$ is the angle between the tangent path of the dome surface and the axis of the mandrel, which can be expressed as follows:

(20) $$\varphi_{1}={tan}^{-1}\left(r^{\prime }\right)$$

**Figure 14:**
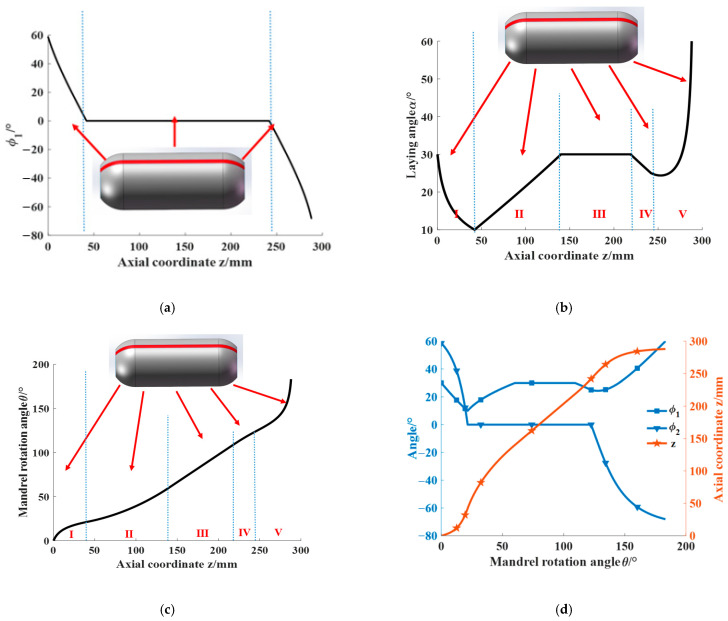
Variation curve of the placement process parameters: (**a**) variation curve of $\varphi_{1}$; (**b**) variation curve of the laying angle α; (**c**) variation curve of mandrel rotation angle θ; (**d**) variation curves for each process parameter based on the mandrel rotation angle θ.

The accuracy of the prepreg’s laying angle plays a crucial role in the AFP process for pressure vessel preparation. The press roller mechanism, controlling the laying angle, moves axially on the mandrel surface within the red area (as shown in Figure 13a) as the mandrel rotates. The laying angle is precisely controlled via the angles $\varphi_{1}$ and $\varphi_{2}$ of the press roller and the mandrel, respectively. Setting the left ellipsoidal dome parameter $\varphi_{1}$ as positive, the change in $\varphi_{1}$ during the laying of the press roller mechanism from left to right is illustrated in Figure 14a. The $\varphi_{1}$ of the cylinder section is 0 since the laying head is parallel to the mandrel axis in that region. The laying angle α, equivalent to $\varphi_{2}$ of the press roller, is shown in Figure 14b for the placement path of the mandrel, which is divided into five areas, namely, I and V (the laying angle change sections of the left and right dome regions), II and IV (the laying angle transition sections of the cylinder section), and III (the cylinder constant laying angle section). Similarly, the mandrel rotation angle change curve corresponding to the direction of the axis length of the prepreg placed from left to right via the press roller is shown in Figure 14c. The change curves of $\varphi_{1}$, $\varphi_{2}$, and the axial displacement of the press roller based on the mandrel rotation angle are illustrated in Figure 14d, which can effectively guide the press roller mechanism and the mandrel rotation axis to coordinate and control the precise and smooth in situ forming of prepreg on the mandrel surface. To validate the placement path planning method, actual laying verification on the machine is necessary, which requires investigating the trajectory of the press roller and the mandrel rotation axis.

**Figure 15:**
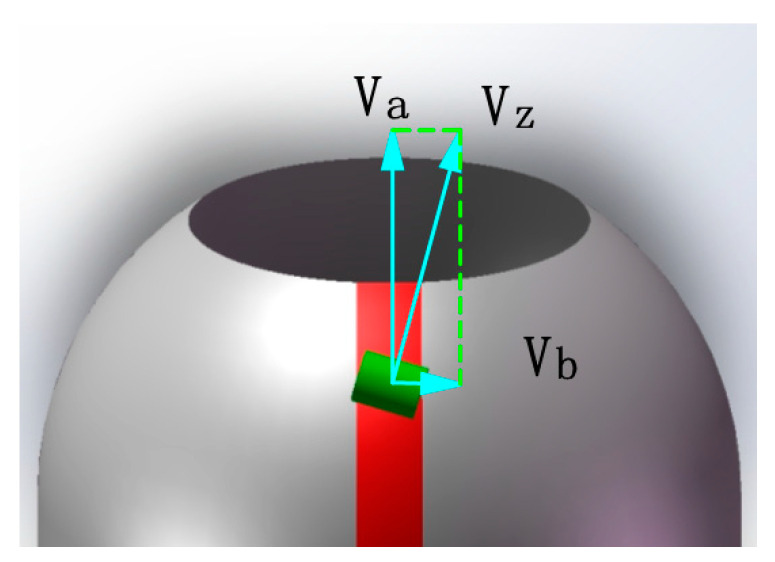
Schematic diagram of the breakdown of the prepreg laying speed.

The laying speed plays a crucial role in the forming quality and efficiency of composite components and is influenced by other process parameters. Thus, studying the stable regulation of prepreg laying speed via the robot and mandrel rotation axis is of great significance in producing high-quality and efficient composite components. As depicted in Figure 15, the prepreg laying speed $V_{Z}$ comprises the axial shift speed of the laying head ($V_{a}$) and the mandrel rotation speed ($V_{b}$). The values of $V_{a}$ and $V_{b}$ can be calculated based on the laying angle at different moments.

**Figure 16:**
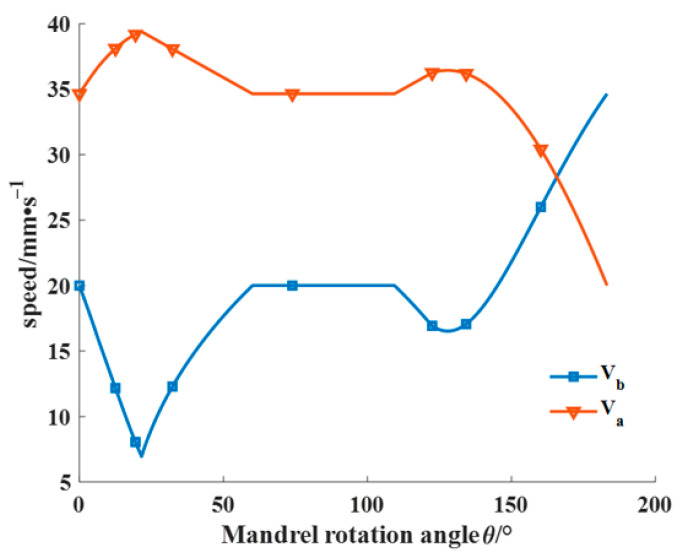
Speed curve of the press roller shift speed and mandrel rotation speed based on the mandrel rotation angle.

The presented Figure 16 illustrates the press roller shift speed and mandrel rotation speed curve when the prepreg laying speed is set to 40 mm/s using the laying angle curve in Figure 14b as a reference. Based on the research findings in this section, the robot and mandrel rotation axis can collaboratively generate G codes, which is used as a common input for the robot control system. Furthermore, the AFP system can be efficiently controlled to prepare pressure vessels of superior quality and productivity by combining the results of placement path analysis with the motion parameter analysis.

## 5. Conclusions

This paper introduces a novel method for calculating the AFP placement path within pressure vessels. The approach is rooted in the wrinkle defect criterion governing prepreg forming on curved surfaces and leverages principles from differential geometry. The goal is to generate placement paths that sidestep both wrinkle and overlap defects. Different from traditional path planning strategies that mainly consider initial paths, the proposed method can directly obtain the laying range in the domain of the rotating surface, determine the placement path by selecting the laying angle, significantly improving the efficiency of placement path planning, reducing the trial-and-error costs, and facilitating the combination with optimization methods for placement path optimization analysis. In addition, the uniform laying envelope shell model of prepreg is simulated to obtain suitable process parameters. Finally, combining the motion characteristics of the AFP equipment, the motion status of the press roller is decomposed to obtain the motion parameters of the press roller, which guides the AFP equipment to complete the laying action.

## Data Availability

All data, models, or code that support the conclusions of this study are available from the corresponding author upon reasonable request.

## References

[1] Lin L., Wang X., Yang B., Zhang L., Zhao Z., Qu X., Lu Y., Jiang X., Lu S. (2021). Conditionmonitoring of composite overwrap pressure vessels based on buckypaper sensor and MXene sensor. Compos. Commun..

[2] Daghighi S., Weaver P.M. (2021). Three-dimensional effects influencing failure in bend-free, variable stiffness composite pressure vessels. Compos. Struct..

[3] Zu L., Xu H., Jia X., Zhang Q., Wang H., Zhang B. (2020). Winding path design based on mandrel profile updates of composite pressure vessels. Compos. Struct..

[4] Li H., Li M. (2022). Constant Winding Angle Curve on Revolution Surface and its Application. Comput. Des..

[5] Xiao R., Shi J., Xiao J. (2021). Study of Allowable Interlaminar Normal Stress Based on the Time–Temperature Equivalence Principle in Automated Fiber Placement Process. Polymers.

[6] Jiang J., He Y., Wang H., Ke Y. (2021). Modeling and experimental validation of compaction pressure distribution for automated fiber placement. Compos. Struct..

[7] Matsuzaki R., Mitsui K., Hirano Y., Todoroki A., Suzuki Y. (2021). Optimization of curvilinear fiber orientation of composite plates and its experimental validation. Compos. Struct..

[8] Yan L., Chen Z.C., Shi Y., Mo R. (2014). An accurate approach to roller path generation for robotic fibre placement of free-form surface composites. Robot. Comput. Manuf..

[9] Bruyneel M., Zein S. (2013). A modified Fast Marching Method for defining fiber placement trajectories over meshes. Comput. Struct..

[10] Wang X., An L., Zhang L., Zhou L. (2008). Uniform coverage of fibres over open-contoured freeform structure based on arc-length parameter. Chin. J. Aeronaut..

[11] Shirinzadeh B., Cassidy G., Oetomo D., Alici G. (2007). Trajectory generation for open-contoured structures in robotic fibre placement. Robot. Comput. Manuf..

[12] Zhao C., Xiao J., Huang W., Huang X., Gu S. (2016). Layup quality evaluation of fiber trajectory based on prepreg tow deformability for automated fiber placement. J. Reinf. Plast. Compos..

[13] Wehbe R., Tatting B., Rajan S., Harik R., Sutton M., Gürdal Z. (2020). Geometrical modeling of tow wrinkles in automated fiber placement. Compos. Struct..

[14] Belhaj M., Hojjati M. (2018). Wrinkle formation during steering in automated fiber placement: Modeling and experimental verification. J. Reinf. Plast. Compos..

[15] Brasington A., Sacco C., Halbritter J., Wehbe R., Harik R. (2021). Automated fiber placement: A review of history, current technologies, and future paths forward. Compos. Part C Open Access.

[16] Zhang L., Wang X., Pei J., Zhou Y. (2020). Review of automated fibre placement and its prospects for advanced composites. J. Mater. Sci..

[17] Mu Z., Xu W., Liang B. (2017). Avoidance of multiple moving obstacles during active debris removal using a redundant space manipulator. Int. J. Control Autom. Syst..

[18] Doan N.C.N., Lin W. (2017). Optimal robot placement with consideration of redundancy problem for wrist-partitioned 6R articulated robots. Robot. Comput. Manuf..

[19] Wu B., Zhang D., Luo M., Zhang Y. (2013). Collision and interference correction for impeller machining with non-orthogonal four-axis machine tool. Int. J. Adv. Manuf. Technol..

[20] Belnoue J.P.-H., Mesogitis T., Nixon-Pearson O.J., Kratz J., Ivanov D.S., Partridge I.K., Potter K.D., Hallett S.R. (2017). Understanding and predicting defect formation in automated fibre placement pre-preg laminates. Compos. Part A Appl. Sci. Manuf..

[21] Brampton C.J., Wu K.C., Kim H.A. (2015). New optimization method for steered fiber composites using the level set method. Struct. Multidiscip. Optim..

[22] Zucco G., Rouhi M., Oliveri V., Cosentino E., O’higgins R.M., Weaver P.M. (2021). Continuous tow steering around an elliptical cutout in a composite panel. AIAA J..

[23] Gurdal Z., Tatting B., Wu K. Tow-placement technology and fabrication issues for laminated composite structures. Proceedings of the 46th AIAA/ASME/ASCE/AHS/ASC Structures, Structural Dynamics and Materials Conference.

[24] Debout P., Chanal H., Duc E. (2011). Tool path smoothing of a redundant machine: Application to Automated Fiber Placement. Comput. Des..

[25] Qu W., He R., Cheng L., Yang D., Gao J., Wang H., Yang Q., Ke Y. (2021). Placement suitability analysis of automated fiber placement on curved surfaces considering the influence of prepreg tow, roller and AFP machine. Compos. Struct..

[26] Zu L., Xu H., Wang H., Zhang B., Zi B. (2019). Design and analysis of filament-wound composite pressure vessels based on non-geodesic winding. Compos. Struct..

[27] Zu L., Xu H., Zhang Q., Jia X., Zhang B., Li D. (2019). Design of filament-wound spherical pressure vessels based on non-geodesic trajectories. Compos. Struct..

[28] Zhang P., Sun R., Zhao X., Hu L. (2015). Placement suitability criteria of composite tape for mould surface in automated tape placement. Chin. J. Aeronaut..

